# Draft Genome Sequence of Bacillus sp. Strain YSP-3, a Halophilic, Alkaliphilic Bacterium Isolated from a Salt Lake

**DOI:** 10.1128/MRA.00882-18

**Published:** 2018-08-23

**Authors:** Shuangyan Wang, Luna Dong, Baisuo Zhao, Shuxia Xu, Kun Wu, Haisheng Wang

**Affiliations:** a College of Life Science, Henan Agricultural University, Zhengzhou, People’s Republic of China; b Graduate School of Chinese Academy of Agricultural Sciences, Beijing, People’s Republic of China; University of Maryland School of Medicine

## Abstract

The halophilic, alkaliphilic bacterium Bacillus sp. strain YSP-3 was isolated from a salt lake. It grows optimally at 8% (wt/vol) NaCl (pH 9.0). The draft genome is composed of 4,006 predicted genes. Genomic analysis showed that various genes are potentially involved in the adaptation mechanisms for osmotic stress and pH homeostasis.

## Abstract (precis)

The halophilic, alkaliphilic bacterium Bacillus sp. strain YSP-3 was isolated from a salt lake.

## ANNOUNCEMENT

The halophilic and alkaliphilic bacterium Bacillus sp. strain YSP-3 was isolated from a salt lake in Jilin, China. In sodium bicarbonate-buffered medium (1), growth occurred at 1% to 13% (wt/vol) NaCl (optimum, 8% NaCl), pH 8.0 to 10.5 (optimum, 9.0), and 15°C to 45°C (optimum, 35°C). Pairwise sequence similarities of 16S rRNA genes were calculated using a global alignment algorithm implemented by applying the EzBioCloud server (2). The result revealed that YSP-3 had the highest 16S rRNA gene sequence similarities with Bacillus aurantiacus K1-5^T^ (97.9  %) and Bacillus populi FJAT-45347^T^ (97.01%). A phylogenetic tree was reconstructed by a neighbor-joining (3) method using MEGA 7 (4). The result showed that YSP-3 formed a distinct phyletic group with B. aurantiacus K1-5^T^ and B. populi FJAT-45347^T^ (data not shown), which revealed that YSP-3 belonged to the genus Bacillus. To understand the adaptive strategies for survival under hypersaline conditions, draft genome sequencing of Bacillus sp. YSP-3 was performed using an Illumina platform.

Total genomic DNA (2 μg) was isolated using a DN12 microbial DNA isolation kit (Aidlab Biotech, Beijing, China) according to the manufacturer’s instructions. A library for genome sequencing was constructed using the NEBNext Ultra DNA library prep kit for Illumina (5). The genome sequence was obtained using an Illumina HiSeq 4000 sequencing platform with a paired-end read length of 2 × 150 bp at approximately 240× coverage and *de novo* assembled using MicrobeTrakr plus v. 0.9.1 (http://microbetracker.org/). Genome annotation was processed using NCBI PGAP (www.ncbi.nlm.nih.gov/genome/annotation_prok). The total length of the draft genome sequence was 4,047,843 bp and yielded 12 contigs, with a G+C content of 48.26%. Among the predicted 4,006 genes, 3,910 putative protein-coding genes (CDSs) were identified. Furthermore, 87 complete RNAs, including 10 rRNAs (8 5S RNAs, 1 16S RNAs, and 1 23S RNA), 72 tRNAs, and 5 noncoding RNAs (ncRNAs) were found.

Analysis of the genome sequence revealed that Bacillus sp. YSP-3 contains 1 gene cluster (*ectA*, *ectB*, and *ectC*) for ectoine biosynthesis from aspartate semialdehyde, 1 *glnA* gene for l-glutamine biosynthesis from l-glutamate, 9 genes of glycine/betaine ABC transporter, 3 genes of Na^+^/solute symporter, 3 genes of Na^+^/alanine symporter, and 2 genes of Na^+^/proline symporter, indicating that Bacillus sp. YSP-3 may resist osmotic stress when salinity increases by taking up compatible solute (i.e., betaine and amino acid) into the cell from the extracellular environment (6, 7). The presence of 6 genes involved in the K^+^ uptake system also implies that strain YSP-3 possibly gains isosmotic cytoplasm though K^+^ as an osmolyte via a "salt-in strategy" when coping with a rapid osmotic shock (8). Alkaliphilic Bacillus sp. YSP-3 contains nine Na^+^/H^+^ antiporter genes (9), a cation/H^+^ antiporter gene (10), and a K^+^/H^+^ antiporter gene (8), which may allow Bacillus sp. YSP-3 survival in alkaline environments (11). These predicted genes provide valuable insights into the adaptive strategies of osmotic balance and Na^+^ homeostasis under salinity stress.

### Data availability.

The draft genome assembly of Bacillus sp. YSP-3 has been deposited at DDBJ/ENA/GenBank under the accession number PDOF00000000.
